# A Clinical Lecture on Cancer of the Tongue

**Published:** 1896-11-28

**Authors:** W. G. Spencer

**Affiliations:** Surgeon to out-Patients and Throat Department, Westminster Hospital.


### A CLINICAL LECTURE ON CANCER OF THE TONGUE.

By W. G. Spencer, Surgeon to Out-patients and Throat Department, Westminster Hospital.

The points concerning cancer of the tongue to which I shall allude in this lecture are those to which my attention has been directed by my own cases, all of which have suffered from the common kind, viz., squamous-celled carcinoma.

**Causation.**—Of the predisposing cause which is the chief factor we know very little. The cancer is far more frequent in men, and the frequency increases with age whilst the malignancy decreases. The question of inheritance is very perplexing; too much ought not to be made of the previous occurrence of cancer in the patient's family, lest thenceforward a needless source of anxiety be laid upon the descendants. Direct inheritance, however, of a cancer diathesis may be the only explanation, as in the case of a most healthy-looking farmer's daughter, acting as a school teacher, and aged twenty-two, who, within two months, developed an extensive cancer of half the tongue and of the glands in the neck. Her father had died of cancer of the throat eight years before. Bad teeth and syphilis form the chief exciting or localising causes. I doubt whether tobacco can act alone as an exciting cause. Smoking, of course, aggravates an ulcer, and common prudence dictates an abstention from tobacco whilst there is a sore of any kind in the mouth. Three out of four women with cancer of the tongue had certainly never smoked. One, an Irish woman, was a smoker; of the three others, one has been referred to, one had neglected a gumma, which finally became malignant, and one had chronic superficial glossitis for four years before the malignant ulcer began, there being no evidence of syphilis, and the glossitis being, apparently, connected with gastritis and a fondness for hot curries. Furthermore, amongst the men were some who had smoked so little that it could not have affected the disease.

**Diagnosis.**—The success of the treatment of cancer of the tongue hangs entirely upon an early diagnosis. The only real difficulty is in distinguishing syphilitic ulceration from cancer. Tubercular ulceration may be dismissed by saying that it is either secondary to pulmonary disease or occasionally is seen as lupus in young people before or about puberty. A simple ulcer, due to a sharp tooth, salivary calculus, or ill-fitting tooth-plate, should show signs of healing within a week of the removal of the cause. A primary syphilitic sore on the tongue is often spoken of, but is really a rare phenomenon, and the absence of the characteristic signs, viz., the rapid development of the swelling and of the glands, together with the rash on the trunk, should prevent the question from being raised. It was, nevertheless, held in the case of a man of twenty-eight that his condition was due to primary syphilis on account of his age, although no rash nor rapid enlargement of glands had occurred. I prepared a microscopic specimen from the surface of the growth, which clearly showed epitheliomatous nest cells, yet, unfortunately, my diagnosis was not agreed to until the disease had got beyond the range of an operation. Both chronic superficial glossitis and an ulcer on the side of the tongue, syphilitic in origin, may pass over into cancer; hence uncured cases require the closest attention. If mercury, given internally, does no good in a week it should be rubbed in until the gums become sore, and so the full effect of the drug quickly reached, and an improvement, if the disease is syphilitic, will be noticeable within a fortnight. At the same time carious and sharp teeth may be removed, smoking stopped, and chromic acid, 10 per cent., may be applied by the patient, or bicyanide of mercury, 1 in 1,000, by the practitioner himself. The most difficult condition to distinguish from cancer is a tertiary syphilitic ulceration, around which there may be extensive induration and some tenderness, or even enlargement of the glands, especially when the mouth is foul. Full doses of iodide of potassium should be given in addition to the mercury, and the dose be quickly raised to thirty grains, or even one drachm, three times a day, whilst the patient meanwhile is kept quiet and is well fed. Ten days of such active treatment will certainly suffice to produce an improvement in a gummatous ulcer, but this improvement must be manifest to the practitioner, and not depend upon the patient's symptoms. Treatment beyond a fortnight in the absence of improvement only wastes precious time and weakens the patient. In any case of doubt a piece is snipped off for microscopic examination after painting 20 per cent. coccaine upon the spot.

**The Operation.**—(1) When the cancer is confined to the dorsum of the tongue, and has not yet invaded the floor of the mouth nor caused an enlargement of glands, the portion of the tongue involved is cut away with scissors. The essential preliminary to the operation is a knowledge on the part of the surgeon of the course of the lingual artery when the tongue is drawn out of the mouth. The artery runs from the great cornu of the hyoid bone to the tip of the tongue in an arched course, which approaches more and more to a straight line as the tongue is drawn out. It runs just below the main muscular mass of the tongue, surrounded by connective tissue and accompanied by the nerve, being separated from the middle line by the vertical fibres of the genio-hyoglossus muscle. The chief point of the operation is to ligature or clamp the artery before division. The patient is anæsthetised by nitrous oxide gas and ether, and then chloroform is given in small quantities through a tube. The mouth is held well open by a gag, which an assistant keeps from slipping, and holds the head so that the mouth shall be in a good light. The head may be let down lower than the trunk, but this is unnecessary if the surgeon is sure of controlling the artery. Two needles are threaded with silk, and if the proposed section is across the whole tongue one is inserted into the tip for traction during the operation, and one into the stump behind the line of section in case it should need to be drawn forwards. If only one side is to be removed, the silk is inserted through the tip one on either side of the middle line. The surgeon draws on the tongue, and keeping close to the jaw divides the frænum and the mucous membrane of the floor as far back as the line of section. If the disease is limited to one side the tongue is split down the middle by first cutting through the epithelium exactly along the middle line with a knife, after which the two halves separate at the raphe merely by the pressure of the fingers. The transverse section is made by short snips a finger's breadth behind the extreme limit of the disease, the dorsalis linguæ at the upper and outer angle and any other vessel being clamped. When the main muscle has been cut through, the lingual artery may be seen and felt enclosed in whitish connective tissue; it is drawn out a little, and ligatured on the face of the stump without including any muscle in the ligature. If little cuts be made, a small nick into the artery will not matter, as the artery can easily be seized, and tied behind the clamp. It is an error hastily to cut the artery clean across, for it retracts, and the blood reflected from the teeth flows back into the pharynx. Should this by chance take place the head should be immediately lowered below the level of the trunk, so that the blood may run out of the nose instead of into the larynx, and the finger be passed back to the hyoid bone, and then hooked outwards towards the angle of the jaw, so as to compress the artery. The mouth and pharynx can now be sponged out, and the artery seized, as it begins to bleed when the compressing finger is relaxed. (2) When the cancer forms an ulcer on the side of the tongue and extends to the floor of the mouth the glands are very early involved. Although clinically there may be nothing more than tenderness, yet microscopical infiltration of the glands can be shown. It is absolutely necessary, therefore, to remove the glands and lymphatics connected with the disease, for otherwise the cancer will continue to grow in the glands which are left behind. This is done by making a submaxillary incision down the inner border of the sternomastoid, from the level of the angle of the jaw to the great cornu of the hyoid bone, thence along the hyoid bone to the middle line, and so vertically upwards to the point of the chin. The flap of skin thus outlined is turned up on to the face, and the lingual artery tied either through the digastric triangle, or behind it just above the great cornu of the hyoid bone. When the submaxillary gland is hooked up the facial artery is straightened out, and brought into view; its proximal end is tied in front of the posterior belly of the digastric muscle and its distal end on the masseter above the submaxillary gland. The corresponding veins are also tied, generally before the arteries. Next the deep fascia and then the mylohyoid muscle are cut away from the lower jaw from the point of the chin back to the angle, so that the month is opened from below. Through this opening, after splitting the tongue, the affected half is drawn out, and cut off without any hæmorrhage, attached to the mylohyoid, the submaxillary salivary and lymphatic glands and deep fascia, the whole forming one mass. There is no need to divide the jaw unless, indeed, a portion has to be excised because it is involved in the cancer. A preliminary tracheotomy is also unnecessary, as no blood need get into the pharnyx at all. I have had to extend this operation—(*a*) To the whole tongue, by repeating the process on the opposite side; if the whole is taken away the silk ligature must be passed through the epiglottis; (*b*) the sternomastoid was retracted, or a vertical incision carried down the line of the carotid, and so other enlarged glands were exposed and removed; (*c*) a piece of the tonsil and soft palate was cut away.

**After-treatment.**—After ligature of small bleeding points, iodoform, of which the largest crystals should be used, is scattered over the cut surface and pressed into the interstices by the finger. If there is still oozing, a strip of iodoform gauze may be inserted, and one end brought out of the angle of the mouth and fixed on the cheek by strapping. The submaxillary wound is sewn up by a continuous suture, except for a small opening at the lowest point, into which a short piece of drainage tube or a little strip of gauze may be inserted. The patient is laid well over on the side with one small pillow, and under his cheek a pad of gauze or towel, so that everything shall run out of the mouth. Whenever mucus collects the nurse inserts a small sponge on a holder, keeping it well against the palate, so as to avoid contact with the stump, and without pushing it back so far as to touch the back wall of the pharynx, rotates the holder, and winds up the mucus on to the sponge. The thread in the stump should not be pulled on except there is respiratory difficulty, which is unlikely to occur unless mucus be allowed to collect. A nutrient enema is given soon after the operation, and repeated every three hours until the patient can take food; suppositories and brandy may be likewise administered. As soon as the patient has recovered from the anæsthetic he is fed with milk, eggs, and meat extracts through a tube, passed behind the stump between the fauces. The amount of food given can hardly be overdone, and it should contain plenty of soluble albumen; the simple cold and hot water extracts of meat, called beef tea, are greatly lacking in this respect. Solids are given as soon as possible; pounded meat may be rolled into small balls with mashed potatoes, smeared with butter, and placed behind the stump by means of a small spoon or fork, fitted on a long, straight shank. The stump is kept covered with crystals of iodoform sprinkled on by the light of a head mirror. The mouth need not be washed out for a day or two; it is then frequently syringed with permanganate of potash. When the mucus becomes ropy, a saline wash consisting of sodium chloride, sodium carbonate, borax, and sugar may be used; and to relieve smarting and heat in the stump during scarring, a solution containing chlorate and nitrate of potash may be employed. The drain opening in the neck tends to prevent any collection of discharge, and it closes before the third week. The removal of glands leaves a depression in the mouth, which has to be kept clear of food, &c.; this afterwards fills up level, especially when the patient begins to talk.

**Complications.**—The one which requires the most care to avoid is that of a failure of general nutrition and loss of appetite, whether due to the pain, exhaustion, and difficulty in swallowing before the operation, or following the operation, with the addition of depression of mind from inability to speak. Nutrient enemata and suppositories should be continued until the patient can talk well; the patient, especially if a man, should get up any time after the first day and move about a little; swallowing is much improved by sitting up, and the wound is in no way disturbed, for the neck is held stiffly. The nurse must attend to the signs or the writing of the patient. Fluid flood often sets up flatus and constipation, and the patient feels much better for regular aperients. Patients after such operations require, if anything, overfeeding. Septic pneumonia is unlikely to occur if the above operation and after-treatment are followed. If the patient has already bronchitis, there is danger of increase after operation, in spite of all precautions, and the patient and his friends must be warned beforehand of the increased gravity of the operation. Hæmorrhage is prevented during the operation, and does not occur later as long as there are no sloughs. In one case, however, I had the misfortune, when removing the anterior half of the tongue through the mouth, to use some friable ligatures, and I broke three in attempting to secure the artery; whilst in the fourth some muscle was included which later on formed a slough. The man lost a pint of blood on the seventeenth day after the operation. I ligatured both linguals in the neck, after which there was rapid healing. The patient is following his previous occupation, and can speak very well.

**Prognosis.**—When the disease is still confined to the dorsum of the tongue a cure may be confidently anticipated. When the ulcer has invaded the side of the tongue and floor of the mouth a cure may be expected, provided that the glands and lymphatics are also taken away. A woman of whose tongue and submaxillary gland I made microscopic specimens has had no return after seven years. With regard to the prognosis after the more extensive operations, one may say that the success of the operation dates from the convalescence of the patient; for by this time he would be at death's door had the operation not been done. Every month, then, or every year after recovery from an extensive operation, is so much added to life.

**Inoperable Cancer.**—Cancer should not be operated upon unless the whole can be completely removed without exposing or resecting the common carotid artery, its bifurcation, the internal carotid artery, or the vagus nerve. I have not had to resect the internal jugular vein, but I believe that it may safely be done if the artery and nerve are not disturbed. In all doubtful cases the operation should commence with a small exploring incision, made over the most deeply-seated glands, and unless these can be removed the operation should be abandoned. Where no operation can be done the patient is relieved by mouth washes, the application of iodoform, removal of irritating teeth, suitable food, alcohol, morphine, and opium for pain. I have tied both linguals, both because they freely anastome in the cancer, and so arrested hæmorrhage from the growth. I see no use in partial removals of the tongue. I do not think that the resection of nerves are likely to relieve pain, for if the disease is limited it is better removed, and, if extensive resection of one nerve, is not likely to be of service.

